# Age-Related Anabolic Resistance: Nutritional and Exercise Strategies, and Potential Relevance to Life-Long Exercisers

**DOI:** 10.3390/nu17223503

**Published:** 2025-11-09

**Authors:** Íñigo M. Pérez-Castillo, Ricardo Rueda, Suzette L. Pereira, Hakim Bouzamondo, José López-Chicharro, Felipe Segura-Ortiz, Philip J. Atherton

**Affiliations:** 1 Abbott Nutrition, R&D, 18004 Granada, Spain; ricardo.rueda@abbott.com; 2 Abbott Nutrition, R&D, Columbus, OH 43219, USA; suzette.pereira@abbott.com; 3 Abbott Nutrition, R&D, Chicago, IL 60064, USA; hakim.bouzamondo@abbott.com; 4 Real Madrid, Medical Services, 28055 Madrid, Spain; jlchicharro@ext.realmadrid.es (J.L.-C.); fsegura@realmadrid.es (F.S.-O.); 5 Centre of Metabolism, Ageing & Physiology (CoMAP), MRC/Versus Arthritis Centre of Excellence for Musculoskeletal Ageing Research, Nottingham National Institute of Health Research Biomedical Research Centre, School of Medicine, University of Nottingham, Nottingham DE22 3DT, UK; philip.atherton@nottingham.ac.uk; 6 Faculty of Sport and Health Sciences, Ritsumeikan University, Kyoto 603-8577, Japan

**Keywords:** anabolic resistance, aging, master athlete, protein synthesis, nutrition, protein intake, supplementation, training, lifelong exercise, muscle

## Abstract

Anabolic resistance, consisting of a diminished ability of aging muscle to respond to anabolic stimuli such as exercise and protein intake, is a key contributor to age-related declines in muscle mass. However, diseases and lifestyle factors associated with aging, including insulin resistance states, overweight/obesity, persistent inflammation and specifically—as a focus herein—physical inactivity and inadequate dietary protein-intake habits, might interact with chronological impairments in muscle anabolism. In this context, master athletes, as individuals who have engaged in lifelong structured exercise, including regular training and sports participation, offer a valuable model for studying processes of chronological vs. inactivity-related aging. While these lifelong exercisers may present improved body composition parameters and other potential benefits in terms of muscle mass and function, it remains unclear whether exercise practice throughout life can prevent the development of anabolic resistance associated with aging. Albeit limited, evidence has indicated that even in lifelong-trained older individuals there is a blunted post-exercise muscle anabolic response compared to younger athletes. However, there is a paucity of data to systematically understand the differences in postprandial anabolic response to varying protein doses in older vs. young athletes. In lieu of the above, it seems reasonable that master athletes may benefit from increasing protein intake closer to the upper limit of current recommendations (1.6–2.0 g/kg/day). In addition, supplementing their diet with ingredients that have established anabolic potential, including branched chain amino acids (BCAAs) such as leucine, the leucine metabolite β-hydroxy-β-methylbutyrate (HMB), and n3-polyunsaturated fatty acids (n3-PUFA), may potentiate the anabolic response to protein and exercise.

## 1. Introduction

Aging is associated with a progressive loss of muscle mass, strength, and function termed sarcopenia [1]. Decrements in muscle mass can be perceived as early as in the third decade of life [2], and are proposed to be a consequence of long-term negative balance between muscle protein synthesis (MPS) and muscle protein breakdown (MPB) [3], and of allied neurodegeneration [4]. Although the etiology of sarcopenia is complex, blunted MPS responses of older adults to anabolic stimuli (exercise and nutrition), termed “anabolic resistance”, has been proposed as an underlying cause of the loss of muscle mass associated with aging [5]. However, isolating the role of primary aging in the onset of anabolic resistance is challenging as diseases and lifestyle factors, so-called “secondary aging” [6], also contribute to impaired muscle anabolism. These include poor physical activity habits [7], inadequate protein intake [8], muscle disuse [9,10], insulin resistance [11], increased adiposity [12], and persistent inflammation [13].

Exercise training programs are effective in improving insulin sensitivity and body composition, attenuating chronic inflammation, and affording different metabolic benefits in healthy adults [14,15,16]. Master athletes represent the foremost clinical research model of lifelong exercise practice. These are athletes, typically over 35 years old, who train for and compete in organized sports, and follow lifelong-structured exercise routines and healthy dietary patterns recommended for athletes [17]. Characteristics of master athletes, including age and training routine, are dependent on their respective sports disciplines; however, there are documented cases of master athletes exceeding 90 years of age [18]. Contrary to untrained age-matched adults, endurance-trained master athletes display functional and physiological features, such as maximal oxygen consumption (VO_2_ max) rates and body composition, closer to that of untrained young individuals [19]. Similarly, strength/power-trained master athletes possess greater strength than untrained young adults [19]. These functional and physiological improvements are accompanied by preserved muscle capillarization and mitochondrial functional capacity [20]. In light of these studies, some authors have put forth the hypothesis that master athletes, as prime examples of lifelong exercise practice, may not be subject to age-related anabolic resistance in relation to protein intake [21], presumably since this would undermine maintained adaptation. To the contrary, some evidence has indicated that master athletes might not be exempt from age-related anabolic resistance following exercise protocols [22,23], highlighting the need for further research.

Previous reviews on the topic of anabolic resistance have endeavored to summarize the potential mechanisms leading to age-related anabolic resistance, as well as explore interventions aimed to offset anabolic resistance in older adults, particularly in clinical settings [24,25,26]. However, most available research has been conducted either in clinical populations or in older subjects (>60 years old) who are frequently characterized by poor physical activity habits and associated factors (e.g., low-grade systemic inflammation, impaired insulin sensitivity, increased adiposity, etc.), which are often considered contributors to the observed blunted anabolic response. In contrast, master athletes, due to their high and consistent physical activity levels, may offer a clearer lens through which to study the intrinsic effects of aging on muscle anabolism [27]. The aim of the present narrative review is threefold: (1) revisit the available evidence on anabolic resistance associated with aging in both the general aging population and lifelong-trained athletes; (2) delve into potential mechanisms involved in anabolic resistance and examine the role of lifelong exercise; (3) propose potential nutritional strategies to optimize muscle anabolism in aging master athletes, based on available evidence. Throughout this review, master athletes refer to endurance-based sports athletes (>35 years old), unless otherwise specified, while older adults are defined as individuals aged >60 years old. Different age groups (e.g., middle-aged adults) are specified in text when needed. To support a clearer understanding of the literature discussed, Box 1 provides a brief overview of the methodologies commonly used to study muscle protein metabolism in research settings. Detailed methodological overviews of stable isotope labeling for the study of whole-body and muscle specific protein turnover in vivo are available elsewhere [28,29].

Box 1 **Methods for estimating muscle protein synthesis and associated signaling markers.**

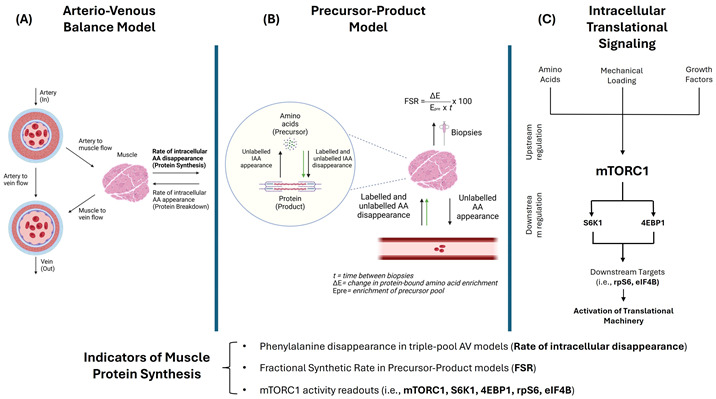
(**A**) Indirect methods for studying muscle protein metabolism mainly include three-pool arteriovenous (AV) balance models, where enrichment in labeled amino acids (AAs) (e.g., phenylalanine (Phe)) in arterial, venous, and intracellular water pools permits calculations of MPS, MPB, and net protein balance aided by measurements of blood flow to the muscle [30,31]. (**B**) Direct assessments of muscle protein turnover can be performed using muscle biopsies through the classic precursor-product model, where infused labeled AAs are the precursor—typically intracellular AA enrichment used as surrogate for amino acyl-tRNA—and proteins are the product. This model, considered the gold standard method for assessing MPS [32], consists of estimating muscle fractional synthesis rates (FSR) of mixed or individual types of proteins (e.g., myofibrillar FSR) through calculating the incorporation of a labeled AA tracer (green arrows) into muscle proteins between biopsies in a given amount of time [33]. Since these methods are more tedious and require maintaining an isotopic steady state, the use of deuterated water (D_2_O) for non-substrate specific ^2^H labeling has garnered great interest as a reliable method for estimating protein turnover over longer periods of time in free-living conditions [34,35]. (**C**) Expression and phosphorylation levels of signaling proteins involved in translational processes are frequently reported to provide insights into MPS. Muscle cell growth signaling is at least partially governed by the serine/threonine kinase mechanistic target of rapamycin (mTOR), with the distinct complex 1 (mTORC1) being responsible for integrating environmental cues to switch between states of anabolism and catabolism [36,37,38]. Inability of older muscles to respond to nutrient sensing and mechanical loading through mTOR signaling pathways has been proposed to underlie age-related anabolic resistance [13,39]. Although not considered “first-line” trigger for initiating muscle hypertrophy, growth factors, mainly insulin and insulin-like growth factor (IGF-1), play important roles in mediating hypertrophic signals through mTOR [37]. Activation of the translational machinery involves mTORC1-dependent phosphorylation of key substrates, including ribosomal protein S6 kinase-1 (S6K1) at Thr389 and eukaryotic translation initiation factor 4E-binding protein 1 (4E-BP1) at Thr37/46. Additional downstream targets such as ribosomal protein S6 (rpS6) and eukaryotic initiation factor 4B (eIF4B), along with phosphorylation of mTOR at Ser2448 and Akt at Thr308, serve as molecular readouts of mTORC1 activity and are frequently reported in studies evaluating MPS responses [40,41]. Figure created with BioRender.com.

## 2. Methodology

A comprehensive, non-systematic literature search was conducted across the scientific databases PubMed (MEDLINE), EMBASE, and the search engine Google Scholar. Combinations of relevant keywords were employed, including but not limited to “anabolic resistance”, “muscle protein synthesis” (MPS), “fractional synthetic rate” (FSR), “mTOR”, “lifelong exercise”, and “master athlete”. Boolean operators (“AND”, “OR”, “NOT”) and wildcards were used in search strings to refine and expand the search scope.

The review included peer-reviewed articles published in English from database inception through March 2025. Titles and abstracts were screened for relevance, and full texts were assessed when necessary. Reference lists of selected articles were manually reviewed to identify additional studies.

Data from clinical studies comparing anabolic responses between young adults and older adults (>60 mean/median years old) were extracted and summarized in Tables S1–S3 in Supplementary S1. These data were used as sources of evidence to inform the narrative synthesis.

## 3. Evidence on Anabolic Resistance Associated with Aging

Early reports suggested the presence of impaired baseline anabolism in fasted older adults at rest, which was not explained by loss of muscle mass and lower fiber size [42,43,44,45]. However, while blunted basal mixed fractional MPS rates in older subjects were also reported in different studies [46,47], a few authors failed to identify such decrements in postabsorptive anabolic capacity following stable isotope labeling [13,48,49,50,51]. Discrepancies were proposed to be caused by the lack of sensitivity of MPB rate assessment methods used before (urinary excretion of 3-methylhistidine in two-pool AV balance models) as well as differences in physical activity habits, among other methodological concerns [52,53]. To date, it is well-accepted that resting/postabsorptive MPS rates are relatively well-maintained with aging, with the focus being placed on the decreased response to anabolic stimuli in older compared to young individuals, termed anabolic resistance [54,55].

### 3.1. Anabolic Resistance to Nutritional Stimuli

To enable comparisons of protein turnover between older and young adults, different forms of nutritional anabolic stimuli have been tested in absence of exercise, including whole food (e.g., beef intake) [56], intake of set quantities of protein isolates [8,57,58,59], oral and intravenous administration of set quantities of AAs [13,48,49,50,51,60,61,62,63], and hyperaminoacidemic clamp [64,65] (Supplementary S1, Table S1). Since phenylalanine (Phe) is an AA that is not synthesized or oxidized in muscle, several of these studies have utilized Phe stable isotopes to evaluate rates of disappearance and appearance in AV balance models [30]. All but one study exploring oral AA/essential amino acids (EAA) provisions in isolation [48] indicated dose- or time-dependent reductions in Phe net balance with age indicating an age-dependent negative impact on muscle protein metabolism [50,51,60]. Using the precursor-product model, similar increases in mixed muscle protein FSR in older and young adults following oral protein and AA intake have been reported by some authors [48,51,56,59,66], while others have documented impaired or delayed mixed, myofibrillar and/or sarcoplasmic fraction MPS in older adults [13,57,62,63], particularly when lower protein/AA doses are provided [8,60].

The co-ingestion of AA or proteins with other nutrients—most notably carbohydrates, which are key stimulators of insulin response—more accurately reflects real-life dietary conditions. Consequently, studies have often assessed anabolic responses using combined AA/protein and glucose intake, or through metabolic clamp techniques such as hyperinsulinemic, hyperglycemic, and/or hyperaminoacidemic clamps [49,57,58,64,65]. While more studies have clearly shown impaired MPS in response to such combinations in comparison with young adults [49,57,64], a few others have reported no differences with age [58,65]. Signaling events are rarely reported in these studies with only a few documenting dysregulation of S6K1 phosphorylation in older individuals subject to hyperinsulinemic and hyperaminoacidemic clamps [64], lower phosphorylation of mTOR, eIF2B, and S6K1 in older men consuming 10 g of EAA [13], and minimal differences in mTOR signaling in older women subject to glucose, insulin, and AA infusions [65] compared to young adults. A systematic review by Shad et al. in 2016 synthesized data on muscle protein fractional synthesis rate (FSR) from several of these studies and suggested that discrepancies in anabolic responses between young and older adults were largely attributable to heterogeneity in the doses and sources of AAs or proteins used [67]. In particular, studies administering 0–40 g of EAAs or high-quality protein, as well as those including additional leucine supplementation, indicated that older individuals may require higher doses of AA/protein to achieve comparable anabolic responses to younger individuals [8,13,60].

### 3.2. Anabolic Resistance to Acute and Long-Term Exercise

Research on age-related anabolic resistance in response to acute exercise in fasted state has mainly focused on bouts of resistance exercise (mainly leg extension series) [39,68,69,70,71], with endurance exercise protocols (e.g., treadmill walking) being less explored [72] (Supplementary S1, Table S2). Results from these studies consistently show blunted MPS response to acute exercise in the fasted state in older adults, whose magnitude seems to depend on exercise intensity, volume and timing. Young adults typically display robust increases in mixed or myofibrillar MPS and activation of relevant anabolic signaling pathways (e.g., mTORC1, S6K1, 4EBP1) following resistance or aerobic exercise, even at moderate intensities or volumes [39,68,70,71,72]. In contrast, older adults often display blunted or delayed MPS [39,71] responses under similar conditions, with some studies indicating that higher exercise volume or intensity is required to elicit comparable anabolic signaling and MPS [70]. Notably, findings are not entirely consistent: Sheffield-Moore et al. reported a more rapid, albeit possibly short-lived, MPS response in older adults compared to younger counterparts [68,72]. Furthermore, changes in anabolic signaling markers do not always align with MPS outcomes, particularly in older individuals [39,69]. This discrepancy suggests a potential disconnect between molecular signaling and functional protein synthesis, which may be attributable to differences in biopsy timing and limitations of cross-sectional analyses of signaling pathways.

More recent studies have taken advantage of classic D_2_O techniques to evaluate the differential anabolic response to longer-term resistance training with aging (Supplementary S1, Table S2). Following three weeks of resistance training consisting of unilateral leg-extension exercise, myofibrillar protein FSR was lower in older compared to young adults [73]. While S6K1 signaling was shown to be impaired in older subjects, signaling responses were not uniform across the exercise program [73]. In the same vein, myofibrillar protein FSR after three sessions of unilateral leg extension and leg press exercises over two days was shown to be lower in older vs. young adults, using D_2_O methods [74]. Thus, limited evidence suggests a blunted anabolic response not only to acute but also to long-term exercise in older individuals. It should be noted that a majority of these studies were conducted in men only, which limits our understanding of anabolic resistance to exercise in older women.

### 3.3. Anabolic Resistance to Combinations of Exercise and Nutrition

**Figure 1:**
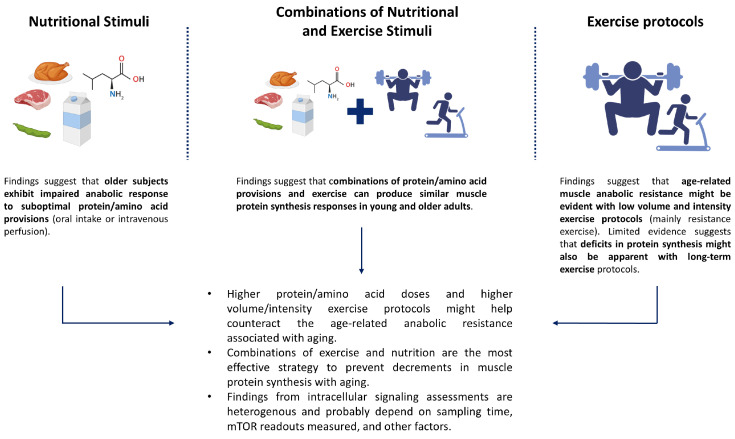
Summary of the evidence on anabolic resistance associated with aging in the general population. Created with BioRender.com.

Resistance exercise in the absence of nutrient provision is insufficient to overcome MPB rates and eventually results in negative net protein balance [32]. Therefore, a combination of nutrition and exercise is optimal for facilitating positive protein balance. Several authors have compared the MPS response of older and young individuals to the combined effect of AA or protein intake and acute resistance or aerobic exercise [66,75,76,77,78,79,80,81,82] (Supplementary S1, Table S3), while others have only reported translational signaling events in similar settings [83,84]. Lower mixed muscle protein FSR in older adults in response to combinations of AA/protein intake with resistance exercise was reported in two studies [76,79], whereas six studies did not report age-related differences in the anabolic response to the combination of dietary AA/proteins with resistance exercise [75,78,81,82], aerobic exercise [77], or both types of exercise [66]. Changes in intracellular signaling pathways are heterogeneously reported, with authors concluding either impaired [75,84], unchanged [77], or even promoted [82] mTORC1 signaling in older individuals in response to the combined effect of exercise and nutrition, which might be attributed to differences in sampling time of the biopsy [76] and training status [83]. A recent study explored how high-volume elastic band exercise in combination with 30 g whey protein impacts the anabolism of older and young males using D_2_O tracer isotope consumption and serial muscle biopsies [80]. During the 48 h post-exercise period, integrated myofibrillar FSR rates were elevated above habitual rates in young but not in older individuals. Although a comprehensive assessment of intracellular signaling protein expression and phosphorylation was conducted, only minor differences in translational efficiency were observed between groups [80]. It should be noted that, with the exception of one study that included women [81], all studies were conducted in males only (Supplementary S1, Table S3). Thus, there is still a gap in the understanding of age-related anabolic resistance to exercise plus nutrition in older women. Overall, some conclusions can be drawn from abovementioned studies. First, studies pooling data from different sources in an attempt to overcome sample size limitations have provided compelling evidence on the presence of anabolic resistance in older individuals (>60 years old) in response to both nutritional and exercise stimuli [8,67]. Second, such age-related impaired response to anabolic stimuli appears to get attenuated when nutritional and exercise interventions are provided in combination [67]. Third, the blunted anabolic response associated with aging seems to be dose-dependent, with higher AA/protein doses and higher exercise volume and intensity frequently correlating with muscle protein synthesis rates similar to those of young adults [8,69,70]. This is aligned with a recent systematic review evaluating postprandial postexercise MPS with dietary leucine, which concluded that older adults are characterized by a slower rate of rise to peak concentration of plasma leucine, greater variability in its magnitude, and a “shift to the right” in muscle anabolic response with increasing leucine doses [85]. Lastly, assessments of intramuscular signaling intermediates have yielded mixed results, which might be consequence of the heterogenous assay methods (e.g., mTOR readouts) and sampling times limited by timing and number of muscle biopsies needed [86]. An overview of these findings is presented in Figure 1.

Evidence on anabolic resistance in middle-aged adults is limited. Specifically, Phillips et al. [87] recruited three cohorts comprising young (18–25 years old), middle-aged (45–55 years old), and older individuals (65–75 years old) who were subject to two acute studies separated by a 20-week whole-body resistance exercise training program. In both acute studies, subjects performed leg extension exercises, were fed an oral nutrition formulation containing 16% protein, and myofibrillar protein FSR were determined. Authors reported blunted hypertrophic responses to the long-term resistance training program in middle-aged and older compared to young adults. While the FSR response to acute exercise and feeding was not different across age groups before the 20-week training protocol, only young individuals displayed higher acute myofibrillar protein FSR after the training program compared to baseline values, indicating that even 20-wk exercise training may not be enough to overcome age-related anabolic resistance [87].

### 3.4. Anabolic Resistance in Master Athletes

Since no study to date has specifically evaluated differential anabolic responses to nutritional stimuli in master athletes, whether lifelong structured exercise can overcome age-related anabolic resistance to nutritional stimuli is yet to be determined. However, a couple of trials have explored aspects of muscle anabolism after exercise protocols in master athletes. Doering et al. [23] compared the MPS rates of master athletes (~53 years old) and young triathletes (~27 years old) performing intense downhill running (damage-inducing exercise) in three consecutive days. Using D_2_O for the analyses, integrated myofibrillar FSR over the 72 h period were calculated under standardized dietary conditions (~1.6 g/kg/day protein), authors observed significantly lower FSR rates in older compared to young athletes [23]. In another study, McKendry et al. [22] enrolled male master athletes (~69 years old) and untrained male older adults (~73 years old) who were provided a bolus of D_2_O, and were asked to complete a series of leg extension exercises under a standardized weight-maintenance diet (17% protein). Integrated myofibrillar muscle protein synthesis rates were calculated over 48 h from values in rested conditions and after leg extension exercise. Also, intramuscular anabolic signaling intermediates were reported at rest, and at 1 h post-exercise. This study found no clear differences in integrated FSR and basal or post-exercise signaling responses between study groups. The authors concluded that, despite long-term history of endurance exercise, master athletes presented a myofibrillar protein synthesis response to unaccustomed resistance exercise similar to that of untrained healthy older adults [22]. Based on this limited evidence, along with findings discussed in the previous section, it seems that signs of impaired muscle anabolism may be apparent already at the fifth decade of life [87], and lifelong endurance exercise might not completely abolish the onset of anabolic resistance to exercise stimuli in older adults [22,23]. Additional research is warranted to understand the impact of lifelong exercise training in the prevention of anabolic resistance associated with aging.

## 4. Factors Contributing to Anabolic Resistance and Their Relevance to Master Athletes

### 4.1. Physical Inactivity

Most studies have observed that 40–80% of adults aged >60 years old do not meet physical activity recommendations [88]. Further, research has suggested that older adults might spend 65–80% of their waking time sedentary [89]. As reviewed in detail elsewhere [86], prolonged bed rest and limb immobilization causes muscle atrophy along with impaired postabsorptive and post-prandial protein synthesis rates [90,91,92,93]. Interestingly, the negative impact of complete muscle disuse on MPS has been reported to be higher in older compared to young individuals [10,94]. Less drastic models of episodic disuse have also been demonstrated to negatively impact anabolism. Specifically, two weeks of 76% reduced daily step-count relative to habitual physical activity was shown to reduce lean mass (4%) and postprandial myofibrillar FSR in healthy older adults (~72 years old) consuming 25 g egg protein [7]. Under similar reduced step-count conditions (70% for two weeks), blunted integrated rates of myofibrillar protein synthesis were reported in overweight older adults compared to baseline values, which were not recovered after 14 days of returning to baseline physical activity [95]. Further, recent research has shown that 91% step count reduction for one week can also reduce myofibrillar FSR in young men (~22 years old), which correlated with downregulated expression of mTOR [96].

These studies have provided increasing evidence linking low physical activity with the onset of anabolic resistance. What is still not clear is whether aging further exacerbates the impact of physical inactivity on anabolic resistance given that most studies comparing muscle anabolic response between young and older adults did not systematically characterize the baseline physical activity status of the enrolled subjects. Only three studies have used either physical activity questionnaires—albeit data were not reported [71]—or wearables to capture habitual physical activity habits, and documented either similar [65] or significantly lower activity [73] in older compared to young adults. In a recent study, Smeuninx et al. reported that a single bout of unilateral leg resistance exercise prior five days of bed rest was sufficient to attenuate a decline in integrated myofibrillar protein synthesis response in well-characterized non-obese physically active older adults (~72 years old) [97]. While endurance master athletes are not completely exempt from having sedentary behaviors [98], it is clear that sports participation and structured exercise contribute to improved physical activity profiles, which can help counteract the deleterious effects of inactivity [99]. Future studies could expand on these findings by exploring how structured exercise patterns maintained throughout life might help distinguish the impact of aging vs. physical inactivity on muscle anabolism. These studies might benefit from recruiting master athletes and older adults with different levels of physical activity to explore whether regular physical activity, independent of competitive sports participation, can confer similar protective effects against anabolic resistance in aging populations.

### 4.2. Compromised Insulin Signaling

Insulin resistance is a hallmark of muscle disuse [100] and has also been observed in non-diabetic older adults [101]. Activation of canonical insulin signaling pathway through insulin or other growth factors, including IGF-1, leads to mTORC1 stimulation through Akt [102]. Although involved in translational efficiency, it should be noted that insulin per se does not stimulate MPS in humans [103]. On the contrary, decreases in insulin signaling translates to blunted Akt inhibition of genes involved in muscle protein breakdown (e.g., atrogenes), particularly ubiquitin proteasome system (UPS) E3 ligases, which may associate with exacerbated MPB [104]. Whether impaired basal and postprandial MPS or increased MPB underlay muscle atrophy in disuse, and the potential contribution of insulin resistance to this process remain far from elucidated [105].

Research in older adults has frequently employed insulin clamp protocols to evaluate the impact of insulin, glucose, and AAs on anabolic resistance. Because insulin is known to downregulate MPB, isolating the specific impact of insulin resistance on MPS requires maintaining plasma AA concentrations—an objective achieved through insulin clamp methods [106]. Early research demonstrated that the combined effects of hyperaminoacidemia and glucose-induced hyperinsulinemia could augment MPS in young, but not in older adults [49]. Clamp studies also showed that there was a blunted insulin effect on whole-body and muscle-specific anabolism that occurs with aging [106], and differences in insulin sensitivity might partially explain divergent results in anabolic resistance research in fed states [13,58,65,76,77]. Since insulin also plays a major role in promoting muscle perfusion to augment transport of AAs to muscle [107,108,109], it is not surprising that insulin resistance may link to anabolic resistance.

Long-term aerobic exercise is associated with enhanced insulin sensitivity to glucose metabolism [110], evidenced from studies with master athletes [111,112,113]. Cross-sectional analyses have observed significantly lower fasting plasma glucose in master athletes, compared to the general population [114], and improved insulin sensitivity compared to untrained old adults [113]. Further, bouts of aerobic exercise have been reported to stimulate microvascular perfusion and delivery of nutrients to skeletal muscle in older adults [9], and lifelong physical activity has been shown to improve insulin signaling and muscle glucose uptake in middle-age individuals [115]. All these findings have led authors to suggest that it is unlikely that master athletes suffer impaired insulin-mediated delivery of AAs to skeletal muscle [21], but this still needs to be systematically studied. Clamp protocols might help discern between the contributions of insulin resistance and physical inactivity to muscle anabolic resistance in older adults with different levels of physical activity.

### 4.3. Adiposity

Changes in body composition with increased adiposity have been proposed to impact whole-body- and muscle-specific protein turnover, yet evidence remains controversial. In this regard, diminished whole-body protein synthesis rates have been reported in middle-aged obese subjects under AA infusion and hyperinsulinemic, euglycemic clamp conditions compared to young lean individuals [116], while no differences were reported in a study exploring responses to hyperaminoacidemia [117]. Under hyperinsulinemic and hyperaminoacidemic clamp, lower mixed muscle protein-, myofibrillar-, and/or mitochondrial FSR have been documented in obese compared to lean subjects in some [118,119] but not in all studies [117,120].

Exploring experimental conditions more similar to normal fed status, impaired myofibrillar FSR was observed in overweight and obese adults following protein-dense food intake in one study [121]. Whereas the combined anabolic effect of resistance exercise and feeding on myofibrillar FSR was shown to be blunted with obesity in some studies [12], different authors have reported similar postprandial myofibrillar or mixed muscle protein FSR in obese and lean adults after ingestion of 25 g whey protein [122], or after resistance exercise in fasted conditions [123]. In an interesting study conducted by Smeuninx et al., the authors reported lower myofibrillar FSR in response to 15 g milk protein isolate relative to postabsorptive values in older compared to young adults, and this response was exacerbated in a group of obese older individuals, thus revealing compounding impact of aging together with obesity on impairing muscle anabolism [124]. Since some studies have documented impaired postprandial anabolism in overweight adults (BMI ~ 27) [121], it seems reasonable to speculate that in studies that have identified anabolic resistance in older adults, higher adiposity in some of the older subjects might have also been a contributing factor beyond age alone [8,13,56,61,63,75,77,83,84,125]. Mechanisms involved in an adiposity-induced anabolic resistance may include accumulation of intramuscular and perimuscular toxic lipid species that may drive secretion of local, paracrine and systemic inflammatory cytokines, which is inherently linked to insulin resistance [126,127].

Master athletes tend to display healthier body composition and body fat percentage than non-trained age-matched peers [19,128], which suggests that the impact of increased adiposity or obesity on anabolic resistance should be minimal in this population. Not surprisingly, recent research comparing relatively large cohorts of young (20–39 years old, *n* = 109), and older (70–89 years old, *n* = 147) athletes, as well as age-matched non-athlete controls, documented far lower prevalence (e.g., 2–3% vs. 19%) of sarcopenic obesity in trained compared to non-trained older individuals [129]. It is also worth noting that endurance-trained athletes typically exhibit higher levels of intramuscular lipid droplets, similar to obese individuals—a phenomenon known as the “athlete paradox” [130]. However, in athletes, these lipid droplets are more likely to be distributed within type-I myofibers and are generally smaller in size. This distribution and size facilitate efficient energy supply through β-oxidation during prolonged exercise [130]. Notwithstanding, evidence suggests that master athletes may also display higher fat mass relative to their younger peers regardless of sport discipline, thus denoting that lifelong exercise, although clearly beneficial for improving body composition, might be insufficient to completely prevent age-related increases in fat accumulation [129]. Based on this evidence, it can be suggested that decreasing adiposity is a mechanism through which lifelong exercise patterns may help counteract anabolic resistance. However, controlling for body composition parameters and body fat distribution appears to be important when investigating the causes of blunted muscle anabolic responses in physically active individuals.

### 4.4. Low-Grade Inflammation

Aging has been associated with low-grade inflammation, denoted by increased circulating levels of inflammatory mediators (e.g., C-reactive protein (CRP), tumor necrosis alpha (TNF-α), interleukin 6 (IL-6), etc.) [131,132,133]. The underlying mechanisms are multifactorial and may include impaired autophagy and inefficient clearance of cellular and molecular debris [134], emergence of a senescence-associated secretory phenotype [135], and shared pathways with obesity—such as intramuscular and perimuscular lipid accumulation (e.g., ceramides)—which have been proposed to mediate the link between inflammation and impaired protein anabolism in older adults [136,137,138,139]. For instance, increased expression of nuclear factor κB (NFκB), a major signaling mediator of the inflammatory responses [140], has been reported in muscle of older individuals displaying anabolic resistance to nutritional stimuli [13,63]. Some authors have also documented decrements in fasted state skeletal muscle protein FSR in older adults associated with higher circulating levels of pro-inflammatory mediators [141,142], which might contribute to attenuated phosphorylation of mTOR regulators [141,143]. Nonetheless, not all studies evaluating postprandial muscle FSR in older adults classified based on the presence of low-grade inflammation, or subject to anti-inflammatory treatments have supported the role of inflammation in age-related anabolic resistance [144,145]. The role of systemic low-grade inflammation and muscle local inflammation in MPS is a topic to be further explored, and novel models of study have emerged in recent years to help delineate it [146].

As demonstrated in a recent meta-analysis, aerobic exercise programs are effective in attenuating markers of inflammation (CRP, TNF-α, IL-6) in healthy middle-aged and older subjects [16]. Whether maintaining exercise training throughout life is effective to prevent low-grade inflammation associated with aging, oftentimes called “inflammaging”, was recently examined in a meta-analysis of available studies comparing master athletes with older untrained and young untrained adults [147]. Main findings suggested that master athletes present a more anti-inflammatory profile denoted by increased levels of IL-10 and attenuated increases in IL-6 and CRP compared to untrained older peers. However, lifelong exercise still presented higher resting levels of IL-6 and TNF-α coupled with lower IL-10 compared to untrained young adults [147]. It should be noted that none of these studies measured age-related muscle anabolic resistance, and findings should be considered with caution due to the potential risk of bias and considerable heterogeneity of the studies assessed. Nonetheless, it may be valuable to analyze inflammatory marker panels in future studies enrolling physically active individuals to better understand the contribution of low-grade inflammation to muscle anabolism.

In summary, evidence from master athletes, as a model of lifelong structured exercise patterns, indicates that these individuals display improved body composition, lower inflammation and better insulin sensitivity compared with non-trained age-matched subjects. This distinction may help better characterize the mechanisms leading to anabolic resistance with age and the role of long-term structured exercise. A brief summary of recommendations for future studies evaluating factors contributing to anabolic resistance in active individuals and master athletes is presented in Table 1. Besides these factors, novel mechanisms have been proposed to contribute to age-related anabolic resistance. For instance, not only blunted translational efficiency but also translational capacity, denoted by impaired ribosomal biogenesis, has been suggested to contribute to the decreased anabolic response and lower sustained hypertrophy associated with aging [75,148]. In addition, the potential contribution of the gut microbiota to muscle anabolism with aging is being increasingly explored in recent years [149,150]. Further, life-long endurance-trained adults might present differentially methylated regions and expressed genes in muscle compared to resistance-trained and untrained subjects, which is proposed to create a “primed” state for anabolic responsiveness [151]. However, the role of epigenetics in anabolic resistance remains largely unexplored and might present a novel avenue to explore in future studies.

## 5. Optimizing Nutrition to Support Muscle Protein Synthesis in Healthy Master Athletes

### 5.1. Protein

Irrespective of the sport discipline, good nutrition, especially protein quality and quantity, plays a crucial role in supporting an athlete’s training and adaptation. A minimum recommended dietary allowance (RDA) of 0.83 g/kg BW/day is recommended for both young and older adults based on nitrogen balance studies [152]. However, higher relative intakes have been recommended by expert scientific organizations for healthy older individuals (>65 years old) (at least 1.0–1.2 g/kg BW/day) [153], and even higher (typically ≥1.2 g/kg BW/day) for athletes in order to address needs pertaining to metabolic adaptation, repair, remodeling and protein turnover [154]. More recently, the International Society of Sports Nutrition recommended 1.4–2 g/kg/day for building and maintaining muscle mass in healthy, exercising individuals [155]. This could be achieved through minimum intakes of 25–30 g of high quality protein per meal, regularly spaced during the day [156]. Absolute and relative protein intakes needed to maximize post-exercise muscle anabolism have been subject to extensive research [157]. It has been suggested that meals containing similar amounts of highly digestible and high quality protein ~20 g/meal for a 65 kg person might be optimal for maximizing myofibrillar protein synthesis after resistance training, while higher amounts might be needed to compensate AA oxidative losses after endurance efforts in young adults [157]. However, a recent comprehensive analysis showed a differential response between 25 g and 100 g of milk protein intake during recovery from resistance exercise in young active men [158]. According to the authors, these results challenge the notion of a limited magnitude and duration of the anabolic response following nutrient stimulus in humans [158]. There also is evidence of increased MPS in response to 40 g vs. 20 g whey protein in resistance-trained young adults [159]. Thus, higher intake (~30 g or more) of protein per meal would be more appropriate for actively exercising athletes.

To date, research suggests that non-trained older adults have increased protein requirements. Approximately 68% higher protein quantities relative to BW were shown to be needed to equally stimulate MPS in older men at rest compared to young men in one study [8]. Further, a mixed meal containing 70 g protein was shown to induce both greater MPS and whole-body protein synthesis response than an isocaloric meal containing 35 g protein in older adults [160], while previous research only found differences at whole-body level in young subjects consuming the same amounts of protein [161]. Notably, when provided as complex meals, these amounts are higher than those previously suggested to maximally stimulate MPS in both young and older men. Given the lack of research pertaining to anabolic resistance in master athletes, previous reviews have applied safety margins to recommend protein intakes of 0.3–0.4 g/kg BW/meal and 0.5 g/kg BW/meal, after resistance and endurance training respectively, for master athletes to address any potential concern with the sufficiency of their intakes [21]. This would translate to 22.5–30 g and 37.5 g per meal for a 75 kg (165 lbs.) master athlete respectively. This approach appears reasonable in most situations and aligns with research showing that high protein intakes (0.6 g/kg BW/meal) attenuate the loss of peak isometric torque and significantly reduce perceived fatigue in endurance master athletes during recovery from a 30-min downhill run, compared to a 0.3 g/kg BW/meal intake [162]. Again, a more systematic dosing study would further enhance these recommendations.

Adhering to recommended intakes may have important practical implications. For example, a cross-sectional analysis reported that master athletes consuming 1.34 g/kg BW/day had greater muscle strength than those consuming 1.21 g/kg BW/day [163]. However, abovementioned recommended intakes are higher than those reported by some endurance master athletes through dietary surveys, and might be challenging to achieve due to the satiating effects of protein [163,164,165]. The use of protein supplements containing highly digestible and high quality protein (e.g., dairy proteins) in between meals or as part of meals might help master athletes achieve the relatively high protein intakes of ≥1.2 g/kg BW/d (e.g., 1.8 g/kg BW/d) that have been proposed to benefit endurance-trained athletes [21].

### 5.2. EAA/Leucine

EAA-containing supplements, particularly with higher leucine content, might also be considered for individuals consuming sub-optimal amounts of proteins, in order to elicit a maximal MPS response at rest or after exercise. This has been supported by several studies that showed increased resting or post-exercise (resistance exercise) myofibrillar MPS rates after acute ingestion of suboptimal amounts of protein (10–20 g) enriched with leucine (1.5–4.2 g dose) compared to similar amounts of non-enriched protein in older subjects [75,166,167]. Similarly, adding leucine to essential AA supplements has been shown to reverse the attenuated resting or post-exercise postprandial MPS responses in older adults in several studies [60,168]. While master athletes concerned with the sufficiency of their protein intake should reasonably prioritize protein sources rich in leucine (e.g., whey protein), further research is needed to evaluate if chronic use of leucine-enriched supplements translate into longer term improvements in muscle mass and strength benefits beyond the benefits of structured exercise [169]. A potential additional benefit of protein/leucine intake is promoting recovery from exercise-induced muscle damage (EIMD) [170]. It should be noted that, while recovery from EIMD appears to be impaired or delayed with aging [162,171,172,173], evidence in master athletes is mixed [174], with several recent studies concluding similar recovery profiles compared to younger athletes [175,176].

### 5.3. HMB

The leucine metabolite β-hydroxy-β-methylbutyrate (HMB) has been shown to stimulate MPS as well as reduce MPB in both young and older adults [177,178]. Tied to its anabolic and anticatabolic effects on muscle, several studies have supported the role of HMB in promoting post-exercise recovery [179]. This might be beneficial for master athletes who are interested in shortening time to full recovery from EIMD. A recent umbrella review of systematic reviews concluded that there is high level of evidence to recommend the use of HMB for reducing markers of EIMD (circulating creatine kinase (CK) and lactate dehydrogenase (LDH)) [180]. Besides recovery from exercise, recovery from injury/immobilization may be an issue for master athletes. HMB supplementation has shown promising effects on preserving muscle mass during immobilization and recovery of strength during exercise rehabilitation [181]. Accordingly, some authors have proposed that HMB might bring benefits in periods of immobilization to promote MPS in master athletes [182]. Further, HMB supplementation (3 g/day) was documented to improve myofibrillar MPS assessed using D_2_O methods over the first two weeks during a unilateral leg exercise protocol in healthy older men in free-living conditions (~68 years old) [183]. In light of previous reviews supporting the beneficial effects of HMB on preserving lean muscle mass in older adults (≥60–65 years old) [184,185], further studies exploring HMB supplementation in master athletes might expand on the potential role of HMB in addressing anabolic resistance associated with aging, including those with life-long structured training.

### 5.4. Omega-3 Fatty Acids

n3-Polyunsaturated fatty acids (n3-PUFA) are of interest in the context of muscle anabolism and aging, which might also mediate beneficial effects during recovery from exercise [186]. Two studies have reported that 8-week supplementation with n3-PUFA (1.86 g/day eicosapentaenoic acid (EPA) and 1.50 g/day docosahexaenoic acid (DHA)) was associated with enhanced mixed muscle FSR and greater increases in muscle mTOR-S6K1 signaling following hyperinsulinemic and hyperaminoacidemic clamp both in older (~71 years old) [187] and middle-aged subject (~40 years old) [188], compared to corn oil or no supplementation. Although a more recent trial using D_2_O methods documented no additional benefit on MPS with n3-PUFA supplementation (1.86 g/day EPA and 1.54 g/day DHA vs. placebo) during a 6-week progressive unilateral resistance exercise training program in healthy older women (~64–67 years old), a trend toward higher absolute synthetic rate—which informs on the absolute amount of myofibrillar protein synthetized—was shown only with n3-PUFA [189]. Another study reported improved mitochondrial and myofibrillar MPS rates following resistance exercise in older adults allocated to 16-week n3-PUFA supplementation (3.9 g/day, 2.25 EPA:DHA) [190]. These potential effects of n3-PUFA on muscle anabolism are thought to be mediated mainly through their anti-inflammatory effects [190]. However, other studies have reported mixed results or have failed to find any benefit of n3-PUFA on MPS in older individuals [191,192,193]. While uncertainties remain regarding the efficacy of n3-PUFA on improving muscle anabolism with aging, analyses of the literature suggest that these might provide benefits in terms of muscle mass and function in older individuals [194]. Overall, based on the well-established anti-inflammatory properties of *n*-3 PUFAs, their role in downregulating muscle catabolism [195], and their potential beneficial effects on muscle anabolism, master athletes might want to prioritize food sources of n3-PUFA [182].

### 5.5. Optimal Energy Intake

Lastly, the importance of avoiding extended periods of energy restriction should be mentioned. According to a recent systematic review, master athletes have been reported to have equivalent or higher energy intake than national average estimates. For example, master athletes consumed on average 8908 kJ/day compared to 7792 kJ/day in the general population aged over 50 years, while younger master athletes consumed 9073 kJ/day vs. 8872 kJ/day in the general population aged 31–50 years. However, energy intake ranged widely, from 5073 kJ/day to 14,535 kJ/day, suggesting that some master athletes may be at risk of not meeting energy requirements [196]. Caloric restriction is frequently pursued with the purpose of achieving changes in body composition, and can oftentimes occur with intermittent fasting programs, sometimes tied to religious practices [197,198]. However, lower capacity for MPS and decreased translational signaling in fasted and fed states have been observed with modest levels of energy restriction, even when protein intakes are kept constant (1.5 g/kg BW/day) [199]. Table 2 provides a concise summary of nutritional recommendations for master athletes, derived from the evidence discussed above.

## 6. Conclusions

While aging is associated with anabolic resistance, the extent to which this phenomenon is driven by chronological aging vs. modifiable lifestyle factors remains unclear. Master athletes, due to their lifelong engagement in exercise training and competition, offer a unique model to disentangle these influences. However, limited evidence suggests that even in this population, anabolic responses to exercise may be attenuated, indicating that lifelong training may not fully prevent age-related anabolic resistance. Future studies should incorporate postprandial conditions and rigorous methodologies, including detailed characterization of age, exercise and physical activity habits, sport discipline, body composition outcomes, insulin sensitivity, and inflammatory profiles to provide clarity on the mechanisms at play. Importantly, sex-based differences in anabolic responses have been underexplored. Most studies to date have focused on male participants, limiting the generalizability of findings. Future research should consider the inclusion of female participants to better understand potential sex-specific differences and nutritional needs.

Master athletes concerned about their protein intake might consider consuming protein doses closer to the upper range of current recommendations, particularly in endurance-trained athletes, applying safety margins proposed in literature. This may consist of targeted daily intakes of ~1.6–2 g/kg of high-quality protein, distributed across meals providing ~0.3–0.5 g/kg (approximately ~22.5–37.5 g per meal). High-protein supplements in combination with other bioactive ingredients, such as branched-chain AAs (particularly leucine), β-hydroxy-β-methylbutyrate (HMB), and polyunsaturated fatty acids, may help sustain a healthy muscle anabolic response towards improving muscle mass, strength and function in older adults and master athletes.

## Figures and Tables

**Table 1:** Recommendations for research studies evaluating factors contributing to anabolic resistance in active adults and master athletes.

Potential Contributors to Anabolic Resistance	Methodological Considerations in Research Studies
Physical Inactivity	Describe physical activity habits in active individuals (e.g., validated questionnaires, logs, wearables), and sport discipline, years of training history, and participation in competitions in master athletes.
Compromised Insulin Signaling	Consider clamp protocols to isolate the contribution of insulin resistance to muscle anabolic resistance.
Adiposity	Describe body composition parameters and body fat distribution using accurate methodologies (e.g., DXA, MRI).
Low-grade Inflammation	Consider inflammatory marker panels to evaluate systematic inflammatory profiles under resting conditions.

DXA, dual-energy X-ray absorptiometry; MRI, magnetic resonance imaging.

**Table 2:** Summary of nutritional recommendations to support muscle protein synthesis in healthy master athletes.

Nutritional Factor	Recommendation
Protein Intake	Target daily intake of high-quality, leucine-rich protein close to the upper limit of current recommendations (e.g., 1.6–2 g/kg/day), distributed across meals providing 0.3–0.5 g/kg (approximately 22.5–37.5 g per meal) [21].
n3-PUFA	Prioritize dietary sources of EPA/DHA to meet at least established recommendations (250–500 mg/day EPA + DHA) [200].
Other Bioactive Ingredients	Consider supplementation with HMB (e.g., 3 g/day), particularly in situations of episodic disuse or rehabilitation [201].
Energy Intake	Avoid extended periods of restricted energy availability by maintaining adequate daily energy intake (e.g., >30 kcal/kg/LBM) [201].

n3-PUFA, n3-polyunsaturated fatty acids; DHA, docosahexaenoic acid; EPA; eicosapentaenoic acid; HMB, β-hydroxy-β-methylbutyrate; LBM, lean body mass. Recommendations herein presented are safe and apply to healthy master athletes (e.g., lacking pre-existing kidney or liver disease).

## Data Availability

No new data were created or analyzed in this study.

## Supplementary Materials

The following supporting information can be downloaded at: https://www.mdpi.com/article/10.3390/nu17223503/s1, Supplementary S1. Studies exploring anabolic responses in young and older adults: Table S1. Studies exploring anabolic responses to nutritional stimuli in rested young and older adults; Table S2. Studies exploring anabolic responses to exercise in fasted state in older and young adults; Table S3. Studies exploring anabolic responses to combinations of exercise and proteins/amino acids in older and young adults.
