# Supplementary material for: Age-Related Anabolic Resistance: Nutritional and Exercise Strategies, and Potential Relevance to Life-Long Exercisers
Source: Nutrients. 2025 Nov 9;17(22):3503. doi: 10.3390/nu17223503 (PMC12655298; doi:10.3390/nu17223503)
Supplement: Supplementary file 1 [file nutrients-17-03503-s001.zip › nutrients-3961235-supplementary.pdf]

Supplementary S1. **Studies exploring anabolic responses in young and older adults**

Table S1. Studies exploring anabolic responses to nutritional stimuli in rested young and older adults

Authors & Year of Publication	Population of Study	Stable Isotope Protocol	Nutritional Stimulus	Muscle Biopsy Sampling Times	Findings on Anabolic Response
			Studies supporting anabolic resistance		
**Volpi et al., 2000 [1]**	Young: n = 5 (1 female, 4 male) adults; age: 30 ± 2 years old; BMI: 24.7 ± 0.4; physically active.<br><br>Older: n = 5 (1 female, 4 male) adults; age: 72 ± 1 years old; BMI: 25.9 ± 0.9; physically active.<br><br>*(Mean ± SE)*	AV balance and FSR models using L-[ring-$^{2}H_5$] Phe.<br><br>Overnight fasting (*no information on previous exercise*).	Oral AA and glucose mixture (40 g AA & 40 g glucose) in small boluses every 10 min for 3 h.	Muscle biopsies were taken at baseline, and before and after the 3 h AA–glucose administration.	Despite similar AA availability and similar insulin and glucose kinetics, the mixed MPS response in older adults was lower when combined with glucose administration, compared to young.
**Guillet et al., 2004 [2]**	Young: n = 6 adults (*sex not reported*); age: 25 ± 1 years old; BMI: 24.4 ± 1.2; FM: 15.4 ± 2.8%; *physical activity not reported*.<br><br>Older: n = 8 adults (*sex not reported*); age: 72 ± 2 years old; BMI: 26.9 ± 0.8; FM: 23.9 ± 1.9%; *physical activity not reported*.<br><br>*(Mean ± SEM)*	FSR model using L-[1-^{13}C] leucine.<br><br>Overnight fasting (*no information on previous exercise*).	AA mixture and insulinemic clamp protocols.	Muscle biopsies were taken before and after the 4 h insulin and AA infusion.	The effects of insulin and AAs on mixed, mitochondrial, and sarcoplasmic MPS is compromised in older adults.<br>No significant differential changes in the phosphorylation status of Akt, mTOR, 4E-BP1 between both groups were observed, but S6K1 phosphorylation was promoted only in young adults.

**Babraj et al., 2005 [3]**	Young: n = 8 men; age: 28 ± 6 years old; BMI: 24 ± 3; *physical activity not reported.*<br><br>Older: n = 8 men; age: 70 ± 6 years old; BMI: 26 ± 4; *physical activity not reported*.<br><br>(*Mean ± SD*)	FSR model using [1-^{13}C] ketoisocaproate. Octreotide and insulin clamp used to control hormonal responses.<br><br>*Previous fasting or information on exercise not specified.*	Oral EAA mixture (20 g) as single bolus intake.	Muscle biopsies were taken before and 3 h after EAA ingestion.	The nutritional stimulus resulted in significant stimulation of myofibrillar MPS in both groups, but it was significantly lower in older adults.
**Cuthbertson et al., 2005 [4]**	Young: n = 20 men; age: 28 ± 6 years; BMI: 24 ± 3; FM: 18 ± 6%; physically active.<br><br>Older: n = 24 men; age: 70 ± 6 years; BMI: 26 ± 4; FM: 24 ± 5%; physically active.<br><br>(*Mean ± SD*)	FSR model using [1-^{13}C] ketoisocaproate. Octreotide and insulin clamp used to control hormonal responses.<br><br>Overnight fasting (12 h) (*no information on previous exercise*).	Oral EAA mixtures (0–40 g) in bolus doses in dose-response design.	Muscle biopsies were taken before and 3 h after EAA ingestion.	Older adults showed reduced responsiveness of myofibrillar and sarcoplasmic MPS to EAA, independent of insulin or IGF-1, despite higher plasma and intramuscular leucine availability. Reduced phosphorylation of mTOR and S6K1 and increased NF-κB expression in older muscle suggest impaired anabolic signaling.
**Katsanos et al., 2005 [5]**	Young: n = 8 (4 female, 4 male); age: 30.6 ± 2.0 years old; BMI: ~24; FM: 25.2 ± 3.3%; physically untrained.<br><br>Older: n = 11 (4 female, 7 male); age: 67.6 ± 2.0 years; BMI: ~27.1; FM: 33.7 ± 3.0%; physically untrained.<br><br>(*Mean ± SEM*)	AV balance model using L-[ring-$^{2}H_5$] Phe.<br><br>Overnight fasting and no physical exercise in the previous 48 h.	Oral EAA mixture (6.7 g) as single bolus intake.	Muscle biopsies were taken at baseline, before and twice after EAA ingestion (1 h and 3.5 h, respectively).	Diminished muscle protein accretion in response to small EAA doses was reported in older adults, despite similar aminoacidemia and insulin responses compared to young subjects.

**Katsanos et al., 2006 [6]**	Young (26% leucine): n = 8 (4 female, 4 male); age: 30.6 ± 2.0 years old; BMI: ~24.2; FM: 25.2 ± 3.3%; *physical activity not reported.*<br><br>Young (41% leucine): n = 8 (4 female, 4 male); age: 28.8 ± 2.6 years old; BMI: ~26.4; FM: 29.8 ± 2.0; *physical activity not reported.*<br><br>Older (26% leucine): n = 10 (3 female, 7 male); age: 66.7 ± 2.0 years old; BMI: ~27.8; FM: 31.2 ± 2.8%; *physical activity not reported.*<br><br>Older (41% leucine): n = 10 (5 female, 5 male); age: 66.5 ± 2.2 years old; BMI: ~27.3; FM: 31.7 ± 2.2%; *physical activity not reported.*<br><br>(*Mean ± SEM*)	AV balance and FSR models using L-[ring-$^{2}H_{5}$] Phe.<br><br>Overnight fasting and no physical exercise in the previous 48 h.	Oral EAA mixtures (6.7 g) with either 26% or 41% leucine as single bolus intake.	Muscle biopsies were taken at baseline, before and twice after EAA ingestion (1 h and 3.5 h, respectively).	In older adults, only the leucine-enriched (41%) EAA mixture stimulated MPS and improved net Phe balance while both mixtures were effective in young adults, suggesting that higher leucine content is required to overcome anabolic resistance in the elderly.
**Moore et al., 2015 [7]**	Young: n = 65 men; age: 22 ± 4 years; BMI: 25.1 ± 0.7; *physical activity not reported.*	FSR model using primed constant infusion of L-[ring-$^{13}C_{6}$] Phe.<br><br>No exercise in the previous 48	Oral ingestion of 0–40 g high-quality animal-based protein as single bolus. Protein intake normalized to body mass and lean body mass.	Muscle biopsies were taken before and after EAA ingestion (3–4 h post-ingestion).	Older men required higher relative protein intake (~0.40 g/kg body mass) maximally stimulate myofibrillar MPS compared to younger men (~0.24 g/kg body mass).

	Older: n = 43 men; age: 71 ± 1 years; BMI: 25.7 ± 1.0; *physical activity not reported.*<br><br>(*Mean ± 95% CI*)	h (*no data on previous fasting conditions).*			Basal and maximal MPS rates were similar between groups, but older men showed reduced sensitivity to lower protein doses.
**Mitchell et al., 2017 [8]**	Young: n = 8 men; age: 19.7 ± 0.5 years old; BMI: 22.9 ± 0.7; *physical activity not reported.*<br><br>Older: n = 8 men; age: 70.0 ± 0.8 years old; BMI: 25.5 ± 0.4; *physical activity not reported.*<br><br>Older (arginine): n = 8 men; age: 69.2 ± 1.2 years old; BMI: 25.7 ± 0.9; *physical activity not reported.*<br><br>(*Mean ± SEM*)	AV balance and FSR models using primed constant infusion of L-[ring-$^{13}C_6$] Phe.<br><br>Overnight fasting and no strenuous exercise for 48 h.	Oral ingestion of 15 g EAA (all groups) + 3 g L-arginine (older + arginine group only) as single bolus.	Muscle biopsies were taken at baseline, before and three times after EAA ingestion (1.5 h, 3 h, and 4 h post-ingestion).	Myofibrillar MPS increased postprandially in all groups but was significantly greater in young.<br><br>Arginine supplementation enhanced late-phase muscle perfusion in older men but did not improve MPS.
Studies not supporting anabolic resistance					
**Volpi et al., 1999 [9]**	Young: n = 7 (3 female, 4 male) adults; age: 30 ± 2 years old; BMI: 25 ± 1; physically active.<br><br>Older: n = 8 (2 female, 6 male) adults; age: 71 ± 2; BMI: 26 ± 1; physically active.	AV balance and FSR models using L-[ring-$^{2}H_5$] Phe.<br><br>Analysis of splanchnic first-pass extraction using L-[ring-$^{13}C_6$] Phe.<br><br>Overnight fasting (*no information on previous exercise*).	Oral AA mixture (40 g) in small boluses every 10 min for 3 h.	Muscle biopsies taken at baseline, and before and after the 3 h AA administration.	Despite higher first-pass splanchnic extraction of certain AAs in older adults, similar post-prandial mixed MPS responses were observed when compared to young adults.

	(*Mean* ± *SE*)				
**Paddon-Jones et al., 2004 [10]**	Young: n = 6 (4 female, 2 male) adults; age: 34 ± 4 years old; BMI: 22 ± 1; Physically active.<br><br>Older: n = 7 (4 female, 3 male) adults; age: 67 ± 2 years old; BMI: 25 ± 2; physically active.<br>(*Mean* ± *SD*)	AV balance and FSR models using L-[ring-$^{2}H_{5}$] Phe.<br><br>Fasting for 12 h and refrain from strenuous physical activity for 72 h.	Oral EAA mixture (15 g) as single bolus intake.	Muscle biopsies were taken at baseline, immediately before, and 3.5-4 hours after EAA ingestion.	A single bolus of 15 g EAA elicits similar increments in MPS (mixed protein fraction) in young and older adults, but older subjects responded more slowly to the EAA stimulus and remained in positive net balance for a longer time. No change in insulin response to AA intake was observed in older adults.
**Symons et al., 2007 [11]**	Young: n = 10 (5 female, 5 male); age: 41.1 ± 8.0 years old; BMI: 29.5 ± 3.6; FM: 28.5 ± 9.0%; physically active.<br><br>Older: n = 10 (5 female, 5 male); age: 70.2 ± 5.1 years old; BMI: 24.1 ± 2.0; FM: 27.3 ± 5.8%; physically active.<br><br>(*Mean* ± *SD*)	FSR model using L-[ring-$^{13}C_{6}$] Phe.<br><br>Overnight fasting and no strenuous activity for 72 h prior.	113 g lean beef (providing ~10 g EAA) as single meal intake.	Muscle biopsies were taken before and twice after EAA ingestion (1 h and 3.5 h, respectively).	Despite higher plasma EAA concentrations in older adults, the anabolic response was similar (mixed protein fraction), indicating that aging does not impair the acute anabolic response to a protein-rich meal.
**Koopman et al., 2009 [12]**	Young: n = 10 men; age: 23 ± 1 years old; BMI: 22.9 ± 0.6; physically untrained.<br><br>Older: n = 10 men; age: 64 ± 1 years old; BMI: 24.7 ± 0.7; physically untrained.<br><br>(*Mean* ± *SEM*)	FSR model and whole-body AA kinetics using oral L-[^{13}C] Phe-labeled casein and i.v. infusion of L-[$^{2}H_{5}$] Phe, L-[^{13}C] leucine, and L-[$^{2}H_{2}$] tyrosine.<br><br>Overnight fasting and standardized diet; no intense exercise for 72 hours prior.	35 g intrinsically labeled micellar casein protein as single bolus intake.	Muscle biopsies were taken before and 6 h after EAA ingestion.	No significant differences in protein digestion, absorption, splanchnic extraction, or mixed MPS between young and older men.

**Chevalier et al., 2011 [13]**	Young: n = 8 women; age: 23.6 ± 1.1 years old; BMI: 21.1 ± 0.7; FM: 27.2 ± 1.5%; PAL: 1.41 ± 0.06<br><br>Older: n = 8 women; age: 73.0 ± 2.7 years old; BMI: 24.4 ± 0.9; FM: 38.1 ± 1.9%; PAL: 1.49 ± 0.05<br><br>(*Mean ± SEM*)	FSR model using L-[$^2H_5$] Phe.<br><br>Whole-body leucine kinetics using L-[^{13}C] leucine.	Hyperinsulinemic, hyperglycemic, hyperaminoacidemic clamps simulating fed steady state.	Muscle biopsies were taken before and 2 h after EAA ingestion.	Whole-body and mixed MPS increased similarly in both groups.<br>No age-related impairment in insulin signaling or anabolic response under fed-state clamp conditions was shown in healthy, active older women.<br><br>Only minor decrements in 4E-BP1 phosphorylation status occurred in older women during the clamp.
**Pennings et al., 2011 [14]**	Young: n = 12 men; age: 21 ± 1 years old; BMI: 23.4 ± 1; FM: 15.2 ± 0.9; physically untrained.<br><br>Older: n = 12 men; age: 75 ± 1 years old; BMI: 24.9 ± 0.8; FM: 20.1 ± 1; physically untrained.<br><br>(*Mean ± SEM*)	FSR model and whole-body AA kinetics using L-[1-^{13}C] Phe and L-[ring-$^2H_5$] Phe.<br><br>Overnight fasting and no exercise in the previous 72 h.	Oral ingestion of 20 g intrinsically labeled casein protein as single bolus.	Muscle biopsies were taken before and 6 h after EAA ingestion.	No age-based differences in Phe kinetics or FSR were shown.<br><br>Higher plasma insulin response and a more rapid increase in plasma AA was apparent in older adults.
**Kiskini et al., 2013 [15]**	Young: n = 12 men; age: 21 ± 1 years old; BMI: 22.1 ± 0.6; FM: 14.4 ± 0.7%; physically untrained.<br><br>Older: n = 12 men; age: 75 ± 1 years old; BMI: 25.8 ± 0.7; FM: 23.2 ± 1.1%; physically untrained.<br><br>(*Mean ± SEM*)	FSR model using intrinsically L-[1-^{13}C] Phe-labeled casein.<br><br>Overnight fasting, and no strenuous physical activity in the last 48 h.	Oral ingestion of 20 g intrinsically labeled casein protein plus 40 g carbohydrate (50% dextrose, 50% maltodextrin) as single bolus.	Muscle biopsies were taken before and twice after EAA ingestion (2 h and 6 h, respectively).	Despite higher insulin and glucose responses in older men, muscle anabolic response (mixed protein fraction) was not impaired following intake of protein plus carbohydrate.

**Gorissen et al., 2014 [16]**	Young (protein): n = 12 men; age: 21 ± 1 years old; BMI: 21.3 ± 0.7; FM: 15.3 ±1.2%; physically untrained.<br><br>Young (carbohydrate and protein): n = 12 men; age: 20 ± 1 years old; BMI: 22.4 ± 0.7; FM: 15 ±1.1%; physically untrained.<br><br>Older (protein): n = 12 men; age: 74 ± 1 years old; BMI: 24.8 ± 1.1; FM: 20.2 ±1.3%; physically untrained.<br><br>Older (carbohydrate and protein): n = 13 men; age: 76 ± 1 years old; BMI: 25.9 ± 0.7; FM: 22.1 ±1.1%; physically untrained.<br><br>(*Mean ± SEM*)	FSR model and whole-body AA kinetics using L-[ring-$^{2}H_{5}$] Phe and L-[ring-3,5-$^{2}H_{2}$] tyrosine infusion.<br><br>Overnight fasting, and no strenuous physical activity in the last 48 h.	Oral ingestion of 20 g intrinsically labeled casein protein with or without 60 g carbohydrate (50% dextrose, 50% maltodextrin) as single bolus.	Muscle biopsies were taken before and twice after EAA ingestion (2 h and 5 h, respectively).	Carbohydrate co-ingestion delayed protein digestion and absorption but did **not** affect whole-body net protein balance or postprandial mixed MPS in either age group.

AA, amino acids; AV balance model, arterio-venous balance model; BMI, body mass index; CI, confidence interval; EAA, essential amino acids; FM, fat mass; FSR, fractional synthetic rate; IGF-1, insulin-like growth factor-1; MPS, muscle protein synthesis; PAL, physical activity level (score); Phe, phenylalanine; SD, standard deviation; SEM, standard error of the mean; SNP, sodium nitroprusside.

Table S2. Studies exploring anabolic responses to exercise in fasted state in older and young adults

Authors & Year of Publication	Population of Study	Stable Isotope Protocol	Exercise Stimulus	Muscle Biopsy Sampling Times	Findings on Anabolic Response
			Studies supporting anabolic resistance		
**Sheffield-Moore et al., 2005 [17]**	Young: n = 6 men; age: 27 ± 3 years; BMI: ~23.8; FM: 20 ± 1%; physically untrained.<br><br>Older: n = 6 men; age: 67 ± 2 years; BMI: ~27.1; FM: 26 ± 2%; physically untrained.<br><br>(*Mean ± SE*)	AV balance and FSR models using L-[ring-$^{2}H_{5}$] Phe.<br><br>Overnight fasting (*no information on previous exercise*).	Acute exercise: 6 sets of 8 repetitions of leg extension at 80% of 1-RM.	Muscle biopsies taken at rest (baseline) and at 10-, 60-, and 180-min post-exercise.	Resistance exercise only increased mixed MPS in older men at 10 min post-exercise, and in younger men at 180 min.<br><br>Only older men showed increased MPB and a transient positive net Phe balance.
**Kumar et al., 2009 [18]**	Young: n = 25 men; age: 24 ± 6 years; BMI: 23 ± 4; physically active.<br><br>Older: n = 25 men; age: 70 ± 5 years; BMI: 24 ± 2; physically active.<br><br>(*Mean ± SEM*)	FSR model using [1,2-$^{13}C_{2}$] leucine infusion and plasma α-KIC as surrogate precursor.<br><br>Overnight fasting (*no information on previous exercise*).	Acute exercise: Unilateral leg extension at 20–90% of 1-RM. Volume matched across intensities.	Muscle biopsies taken at rest, immediately post-exercise, and at 1, 2, and 4 h post-exercise.	Older men had a blunted mixed MPS (30% decreased FSR AUC) and signaling response (S6K1, 4EBP1) compared to young men.<br><br>Only young men showed a correlation between S6K1 phosphorylation and MPS.
**Mayhew et al., 2009 [19]**	Young: n = 21 (*Sex not specified*); age: 27.9 ± 1.0 years; BMI: ~25; physically untrained.	FSR model using L-[ring-$^{2}H_{5}$] Phe infusion.<br><br>Overnight fasting	Acute exercise: Unaccustomed resistance exercise consisting of 3 sets of 8–12 RM on squat, leg press, and knee extension (the single bout of unaccustomed exercise was followed by 16 weeks of progressive	Muscle biopsies taken at rest, and 24 h post-exercise (first bout).	Translational signaling (Akt, S6K1, RPS6, 4EBP1, eIF4E, eIF4G) was upregulated 24 h after unaccustomed exercise in both age groups. On the other hand, mixed MPS increased 96% in young but not in older adults.

	Older: n = 15 (*Sex not specified*); age: 64.4 ± 0.9 years; BMI: ~26; physically untrained.<br><br>(*Mean ± SE*)	(*no information on previous exercise*).	resistance training (3 days/week), but only to evaluate muscle adaptations).		Despite blunted FSR in older adults, both groups achieved similar hypertrophy and strength gains after 16 weeks.
**Fry et al., 2011 [20]**	Young: n = 16 (8 men, 8 women); age: 27 ± 2 years; BMI: 25.1; physically active.<br><br>Older: n = 16 (8 men, 8 women); age: 70 ± 2 years; BMI: 24.2; physically active.<br><br>(*Mean ± SD*)	FSR model using L-[ring-$^{13}C_6$] Phe infusion.<br><br>Overnight fasting and no exercise in the previous 48 h.	Acute exercise: 8 sets of 10 repetitions at 70% 1-RM leg extension.	Muscle biopsies taken at rest, immediately post-exercise, and at 3, 6, and 24 h post-exercise.	Mixed MPS response and mTORC1 signaling (Akt, mTOR, S6K1, 4EBP1) increased significantly in young at all time points, with a depressed response shown in older adults.<br><br>Positive correlation between mTOR/S6K1 phosphorylation and MPS was shown only in young.
**Kumar et al., 2012 [21]**	Young: n = 12 men; age: 24 ± 6 years; BMI: 22 ± 3; FM: 15 ± 7 kg; physically active.<br><br>Older: n = 12 men; age: 70 ± 5 years; BMI: 23 ± 4; FM: 19 ± 9 kg; physically active.<br><br>(*Mean ± SEM*)	FSR model using [1,2-$^{13}C_2$] leucine infusion and plasma α-KIC used as surrogate precursor.<br><br>Overnight fasting and no exercise in the previous 72 h.	Acute exercise: 3 or 6 sets of unilateral leg extension at 40% or 75% 1-RM.	Muscle biopsies taken at rest, immediately post-exercise, and at 1, 2, and 4 h post-exercise.	In young men, increasing volume had minimal effect on myofibrillar MPS (non-significant increase in FSR AUC vs. 110% in older men at 75% 1-RM)). In older men, doubling volume significantly increased MPS and S6K1 phosphorylation at both intensities, suggesting that older muscles require higher volume to achieve comparable MPS.

**Brook et al., 2016 [22]**	Young: n = 10 men; age: 23 ± 1 years; BMI: 23.6 ± 1; FM: ~14 kg; activity counts: 52,445 ± 7462.<br><br>Older: n = 10 men; age: 69 ± 1 years; BMI: 25.8 ± 1; FM: ~21 kg; activity counts: 72,584 ± 5746.<br><br>(*Mean ± SEM*)	D_2O labeling of alanine to measure cumulative FSR over 6 weeks.<br><br>Overnight fasting before baseline values (free living conditions).	Long-term exercise: 6 weeks of unilateral resistance training (6 × 8 reps at 75% 1-RM, 3x/week).<br><br>Diet intake monitoring through 4-day diet diaries.	Muscle biopsies taken at baseline, 3 weeks, and 6 weeks.	Only young adults showed increased myofibrillar MPS at 3 weeks of training whereas no further change was observed at 6 weeks. Older adults showed blunted hypertrophy and myofibrillar MPS response to RET at all time points.<br><br>Blunted ribosomal biogenesis (RNA:DNA), translational efficiency (S6K1), and hormonal responses (testosterone, IGF-1) in older vs. young.
**Reitelseder et al., 2021 [23]**	Young: n = 8 men; age: 23 ± 3 years; BMI: 24.1 ± 2.5; physically untrained.<br><br>Older: n = 7 men; age: 70 ± 4 years; BMI: 26.5 ± 1.9; physically untrained.<br><br>(*Mean ± SD*)	D_2O labeling of alanine over 2 days during habitual daily living.<br><br>FSR model using L-[ring-$^{13}C_6$] Phe infusion over 4 hours in overnight fasted state.	Long-term exercise: 4 consecutive days of unilateral resistance exercise (leg press and leg extension, 4 sets of 8 reps at 70% 1-RM).	Muscle biopsies taken on day 1, and day 3 (after 4 h tracer infusion).	2-day integrated myofibrillar FSR increased in exercised leg in both young and old with no age difference. In contrast, 4-hour fasted FSR increased only in young, exercised leg. Older adults showed lower MPS response in controlled settings but not during daily living.
			Studies not supporting anabolic resistance		
**Sheffield-Moore et al., 2004 [24]**	Young: n = 6 men; age: 29 ± 2 years old; BMI: ~25.8; FM: 17 ± 2%; physically untrained.	AV balance and FSR models using L-[ring-$^{2}H_5$] Phe.<br><br>Overnight fasting	Acute exercise: 45 minutes of treadmill walking at 40% of peak VO_2.	Muscle biopsies taken at baseline and at 10-, 60-, and 180-min post-exercise.	Moderate-intensity aerobic exercise increased mixed MPS and turnover in both young (10-60 min) and older men (10 min), yet older men displayed a more prolonged response when considering blood flow, MPS and MPB.

Older: n = 6 men; age: 69 ± 1 years old; BMI: ~27.2; FM: 28 ± 2%; physically untrained.<br><br>(*Mean ± SE*)	(*no information on previous exercise*).

AUC, area under the curve; AV balance model, arterio-venous balance model; BMI, body mass index; D2O, deuterium oxide; eIF4E, eukaryotic initiation factor 4E; eIF4G, eukaryotic initiation factor 4G; FM, fat mass; FSR, fractional synthetic rate; KIC, α-ketoisocaproic acid; MPB, muscle protein breakdown; MPS, muscle protein synthesis; mTOR, mechanistic target of rapamycin; mTORC1, mechanistic target of rapamycin complex 1; Phe, phenylalanine; RM, repetition maximum; RPS6, ribosomal protein S6; S6K1, ribosomal protein S6 kinase beta-1; SD, standard deviation; SE, standard error; SEM, standard error of the mean; peak VO_2, peak oxygen consumption.

Table S3. Studies exploring anabolic responses to combinations of exercise and proteins/amino acids in older and young adults

Authors & Year of Publication	Population of Study	Nutritional Stimulus	Exercise Stimulus	Stable Isotope Protocol	Muscle Biopsy Sampling Times	Findings on Anabolic Response
			Studies supporting anabolic resistance			
**Koopman et al., 2006 [25]**	Young: n = 8 men, age: 20 ± 1 years old; BMI: 22.5 ± 1.1; physically untrained.<br><br>Older: n = 8 men, age: 75 ± 1 years old, BMI: 25.7 ± 0.8; physically untrained.<br><br>*(Mean ± SEM)*	Carbohydrate: 0.49 g/kg/h glucose + maltodextrin.<br><br>Carbohydrate + protein + leucine: 0.49 g/kg/h glucose + maltodextrin + 0.16 g/kg/h whey protein hydrolysate + 0.03 g/kg/h leucine.<br><br>Repeated bolus after exercise for 330 min (1.33 mL/kg every 30 min).	Acute exercise: Standardized physical activity protocol: 5 min cycling + 6 sets of 10 reps each of leg press and leg extension at 40–75% 1-RM.	FSR model and whole-body AA kinetics using L-[ring-$^{13}C_6$] Phe and L-[ring-$^{2}H_2$] tyrosine.<br><br>Overnight fasting and no exercise in the previous 72 h.	Muscle biopsies taken at 0- and 360-min post-exercise	The mixture of carbohydrate, protein, and leucine improved whole-body protein balance and increased mixed MPS in both age groups; however, older men had lower absolute synthesis rates both with carbohydrates and the with the combination.

**Drummond et al., 2008 [26]**	Young: n = 7 men; 29.7 ± 1.7 years old; BMI: ~28.4; FM: 30.5 ± 7.8 kg (22.8 ± 2,8%); physically untrained.<br><br>Older: n = 6 men; 70.0 ± 2.1 years old; BMI: ~27.1; FM: 18.8 ± 2.0 (23.0 ± 1.3%); physically untrained.<br><br>*(Mean ± SEM)*	20 g of EAA enriched in leucine (35% of total EAA); ingested 1 h post-exercise.	Acute exercise: 8 sets of 10 repetitions of bilateral leg extension at 70% of 1-RM.	FSR model using L-[$^2H_5$] Phe infusion.<br><br>Overnight fasting and no exercise in the previous 24 h.	Muscle biopsies taken at baseline, 1 h, 3 h, and 6 h post-exercise.	Mixed MPS increased 1-3 h post-exercise in young but not in older men. Both groups showed increased MPS 3-6 h post-exercise.<br><br>Activation of mTOR signaling was similar between groups, but ERK1/2-MNK1 activation was blunted and AMPK phosphorylation elevated in older muscle.
**Durham et al., 2010 [27]**	Young: n = 9 men; age: 30 ± 2 years old; BMI: 27 ± 1; FM: 22 ± 1%; physically untrained.<br><br>Older: n = 8 men; age: 67 ± 2 years old; BMI: 27 ± 1; FM: 24 ± 1%; physically untrained.<br><br>*(Mean ± SEM)*	Continuous infusion of AAs (Premasol 10%) during rest and post-exercise.	Acute exercise: 45 min treadmill walking at ~40% VO_2 peak.	AV balance and FSR models using L-[ring-$^{13}C_6$] Phe infusion.<br><br>Overnight fasting and "regular activities of daily living" the week preceding the study.	Muscle biopsies taken 150 and 30 min before exercise, and at 10- and 180-min post-exercise.	Mixed MPS increased post-exercise in both groups, with no significant age-related difference in absolute FSR. However, older adults showed reduced synthetic efficiency and increased fractional outward transport, indicating anabolic resistance despite equal or greater AA availability.
**Marshall et al., 2023 [28]**	Young: n = 8 men; age: 24.3 ± 3.7 years old; BMI: 24.5 ± 1.9; FM: 25.2 ± 5.9 %; average daily step count: 8,524 ± 3,363.<br><br>Older: n = 8 men; age: 67.7 ± 5.6 years old; BMI: 25.5 ± 2.6; FM: 27.3 ± 7.0	30 g whey protein concentrate ingested immediately post-exercise.<br><br>Standardized diet (~1.24 g/kg/day protein) provided during the study.	Acute exercise: Elastic band resistance exercise. 6 sets of 12 repetitions of knee extension at ~70% 1-RM.	Integrated FSR measured using oral D_2O tracer.	Muscle biopsies taken at baseline, pre-exercise, 1 h post-exercise, and 48 h post-exercise.<br><br>Saliva samples collected daily for body water enrichment.	The exercise protocol increased integrated FSR in young men but not in older men.<br><br>Anabolic signaling (e.g., p-Akt, p-S6K1, p-RPS6) increased similarly in both groups at 1 h post-exercise with minor differences being noted.

	%; average daily step count: 7,594 ± 2,884.<br><br>(*Mean ± SD*)					
			Studies not supporting anabolic resistance			
**Brock Symons et al., 2011 [29]**	Young: n = 7 adults (3 men, 4 women); age: 29 ± 3 years old; BMI: 28.9 ± 2.8; FM: 31.9 ± 3.5%; physically active.<br><br>Older: n = 7 adults (3 men, 4 women); age: 67 ± 2 years old; BMI: 27.1 ± 1.5; FM: 30.7 ± 3.7%; physically active.<br><br>*(Mean ± SEM)*	340 g lean beef (90 g protein) consumed 60 min before exercise.	Acute exercise: 6 sets of 8 repetitions of leg extension exercise at 80% 1-RM.	FSR model using L-[ring-$^{13}C_6$] Phe infusion (correction factor to account for lean beef non-labeled Phe).<br><br>Overnight fasting and no strenuous exercise in the previous 72 h.	Muscle biopsies taken at baseline, pre-exercise, and 5 h post-meal/exercise.	Mixed MPS increased by ~108% in both groups (mixed sex) post-meal and exercise without evidence of age-related anabolic resistance.
**Pennings et al., 2011 [14]**	Young: n = 24 men; age: 21 ± 1 years old; BMI: 22.8 ± 0.5 (exercising), 23.4 ± 1 (rest); FM: 15,9 ± 0.9% (exercising), 15.2 ± 0.9% (rest); physically untrained.<br><br>Older: n = 24 men; age: 74 ± 1 years old; BMI: 23.6 ± 0.6 (exercising), 24.9 ± 0.8 (rest); ± 0.7; FM: 17.3 ± 1.0% (exercising), 20.1 ± 1.0% (rest); physically untrained.	20 g intrinsically L-[^{13}C] Phe-labeled casein protein ingested immediately after exercise or at rest.	Acute exercise: 30 min moderate-intensity protocol consisting of 5 min cycling + 6 sets of 10 reps each of leg press and leg extension at 40–75% 1-RM.	FSR model and whole-body AA kinetics using L-[ring-$^{2}H_5$] Phe.<br><br>Overnight fasting and no strenuous exercise in the previous 72 h.	Muscle biopsies taken at 0 min and 360 min post-drink.	Exercising before protein intake significantly increased mixed MPS in both age groups in men. No age-related differences in digestion, absorption, or MPS response were apparent.

	(Mean ± SEM)					
**Atherton et al., 2017 [30]**	Young: n = 18 men; age: 24 ± 6 years old; BMI: 23 ± 2; FM: 13 ± 5 kg; physically untrained.<br><br>Older: n = 18 men; age: 70 ± 5 years old; BMI: 25 ± 2; FM: 19 ± 8 kg; physically untrained.<br><br>(*Mean ± SEM*)	325 ml Slim-Fast Optima (10 g protein + 24 g carbohydrate) enriched with either 4.2 g leucine or 4.2 g alanine (isonitrogenous control) before exercise.	Acute exercise: 6 sets of 8 repetitions of unilateral leg extension at 75% 1-RM.	FSR model using [1,2-$^{13}C_2$] leucine infusion. Plasma α-KIC used as precursor pool.<br><br>Overnight fasting and no strenuous exercise in the previous 72 h.	Muscle biopsies taken at baseline, immediately post-exercise, and 1, 2, and 4 h post-feeding.	Leucine-enriched protein drink significantly enhanced myofibrillar MPS and S6K1 phosphorylation in both young and older men. No age-related difference in net anabolic response when leucine was added.
**Hermans et al., 2023 [31]**	Young: n = 14 men; age: 24 ± 3 years old; BMI: 24.0 ± 3.3; FM: 21.7 ± 6.2 %; physically active.<br><br>Older: n = 15 men; age: 73 ± 3 years old; BMI: 24.1 ± 2.0; FM: 23 ± 2.8 %; physically active.<br><br>(*Mean ± SD*)	30 g protein provided as quark (291 g), enriched with 2.5% L-[ring-$^{13}C_6$] Phe after exercise.	Acute exercise: Single-legged resistance exercise consisting of 6 sets each of leg press and leg extension (2 sets warm up, and 4 sets of 8-10 repetitions at 80% 1-RM).	FSR model using L-[ring-$^{13}C_6$] Phe infusion.<br><br>Overnight fasting and no strenuous exercise in the previous 72 h.	Muscle biopsies taken at baseline, immediately post-exercise, and 240 min post-quark ingestion.	Quark ingestion increased mixed MPS at rest (~70%) and further during recovery from exercise (~30–40%) in both young and older men. No age-related difference in postprandial mixed MPS response was shown.
**Horwath et al., 2024 [32]**	Young: n = 10 men; age: 22 ± 3 years old; BMI: 23.4 ± 0.8; physically active.<br><br>Older: n = 70 ± 0.3 years old; BMI: 22.7 ± 1.3; physically active.<br><br>(*Mean ± SD*)	240 mg EAA/kg BW post-exercise.	Acute exercise: unilateral leg resistance exercise consisting of 10 sets of 10 repetitions.	FSR model using L-[ring-$^{13}C_6$] Phe infusion	Muscle biopsies taken before exercise, after exercise coinciding with EAA ingestion, and at 60 and 180 min into recovery from exercise.	In lean and healthy physically active old men, no sign of anabolic resistance was shown following unilateral resistance exercise and EAA intake.

1-RM, 1 repetition maximum; AV balance model, arterio-venous balance model; BMI, body mass index; BW, body weight; D_2O, deuterium oxide; EAA, essential amino acids; ERK1/2, extracellular signal-regulated kinase 1/2 ; FM, fat mass; FSR, fractional synthetic rate; KIC, α-ketoisocaproic acid; MNK1, MAP kinase-interacting kinase 1; MPS, muscle protein synthesis; mTOR, mechanistic target of rapamycin; mTORC1, mechanistic target of rapamycin complex 1; Phe, phenylalanine; RPS6, ribosomal protein S6; S6K1, ribosomal protein S6 kinase beta-1; SD, standard deviation; SEM, standard error of the mean; VO_2 peak, peak oxygen consumption.

References

1. Volpi, E.; Mittendorfer, B.; Rasmussen, B.B.; Wolfe, R.R. The response of muscle protein anabolism to combined hyperaminoacidemia and glucose-induced hyperinsulinemia is impaired in the elderly. *The Journal of clinical endocrinology and metabolism* **2000**, *85*, 4481–4490, doi:10.1210/jcem.85.12.7021.
2. Guillet, C.; Prod'homme, M.; Balage, M.; Gachon, P.; Giraudet, C.; Morin, L.; Grizard, J.; Boirie, Y. Impaired anabolic response of muscle protein synthesis is associated with S6K1 dysregulation in elderly humans. *FASEB journal : official publication of the Federation of American Societies for Experimental Biology* **2004**, *18*, 1586–1587, doi:10.1096/fj.03-1341fje.
3. Babraj, J.A.; Cuthbertson, D.J.; Smith, K.; Langberg, H.; Miller, B.; Krogsgaard, M.R.; Kjaer, M.; Rennie, M.J. Collagen synthesis in human musculoskeletal tissues and skin. *American journal of physiology. Endocrinology and metabolism* **2005**, *289*, E864–869, doi:10.1152/ajpendo.00243.2005.
4. Cuthbertson, D.; Smith, K.; Babraj, J.; Leese, G.; Waddell, T.; Atherton, P.; Wackerhage, H.; Taylor, P.M.; Rennie, M.J. Anabolic signaling deficits underlie amino acid resistance of wasting, aging muscle. *FASEB journal : official publication of the Federation of American Societies for Experimental Biology* **2005**, *19*, 422–424, doi:10.1096/fj.04-2640fje.
5. Katsanos, C.S.; Kobayashi, H.; Sheffield-Moore, M.; Aarsland, A.; Wolfe, R.R. Aging is associated with diminished accretion of muscle proteins after the ingestion of a small bolus of essential amino acids. *The American journal of clinical nutrition* **2005**, *82*, 1065–1073, doi:10.1093/ajcn/82.5.1065.
6. Katsanos, C.S.; Kobayashi, H.; Sheffield-Moore, M.; Aarsland, A.; Wolfe, R.R. A high proportion of leucine is required for optimal stimulation of the rate of muscle protein synthesis by essential amino acids in the elderly. *American journal of physiology. Endocrinology and metabolism* **2006**, *291*, E381–387, doi:10.1152/ajpendo.00488.2005.
7. Moore, D.R.; Churchward-Venne, T.A.; Witard, O.; Breen, L.; Burd, N.A.; Tipton, K.D.; Phillips, S.M. Protein ingestion to stimulate myofibrillar protein synthesis requires greater relative protein intakes in healthy older versus younger men. *The journals of gerontology. Series A, Biological sciences and medical sciences* **2015**, *70*, 57–62, doi:10.1093/gerona/glu103.
8. Mitchell, W.K.; Bethan, E.P.; Daniel, J.W.; John, P.W.; Debbie, R.; Jonathan, N.L.; Kenneth, S.; Philip, J.A. Supplementing essential amino acids with the nitric oxide precursor, l-arginine, enhances skeletal muscle perfusion without impacting anabolism in older men. *Clinical Nutrition* **2017**, doi:10.1016/j.clnu.2016.09.031.
9. Volpi, E.; Mittendorfer, B.; Wolf, S.E.; Wolfe, R.R. Oral amino acids stimulate muscle protein anabolism in the elderly despite higher first-pass splanchnic extraction. *The American journal of physiology* **1999**, *277*, E513–520, doi:10.1152/ajpendo.1999.277.3.E513.
10. Paddon-Jones, D.; Sheffield-Moore, M.; Zhang, X.J.; Volpi, E.; Wolf, S.E.; Aarsland, A.; Ferrando, A.A.; Wolfe, R.R. Amino acid ingestion improves muscle protein synthesis in the young and elderly. *American journal of physiology. Endocrinology and metabolism* **2004**, *286*, E321–328, doi:10.1152/ajpendo.00368.2003.
11. Symons, T.B.; Schutzler, S.E.; Cocke, T.L.; Chinkes, D.L.; Wolfe, R.R.; Paddon-Jones, D. Aging does not impair the anabolic response to a protein-rich meal. *The American journal of clinical nutrition* **2007**, *86*, 451–456, doi:10.1093/ajcn/86.2.451.
12. Koopman, R.; Walrand, S.; Beelen, M.; Gijsen, A.P.; Kies, A.K.; Boirie, Y.; Saris, W.H.; van Loon, L.J. Dietary protein digestion and absorption rates and the subsequent postprandial muscle protein synthetic response do not differ between young and elderly men. *The Journal of nutrition* **2009**, *139*, 1707–1713, doi:10.3945/jn.109.109173.
13. Chevalier, S.; Goulet, E.D.; Burgos, S.A.; Wykes, L.J.; Morais, J.A. Protein anabolic responses to a fed steady state in healthy aging. *The journals of gerontology. Series A, Biological sciences and medical sciences* **2011**, *66*, 681–688, doi:10.1093/gerona/glr036.

14. Pennings, B.; Koopman, R.; Beelen, M.; Senden, J.M.; Saris, W.H.; van Loon, L.J. Exercising before protein intake allows for greater use of dietary protein-derived amino acids for de novo muscle protein synthesis in both young and elderly men. *The American journal of clinical nutrition* **2011**, *93*, 322–331, doi:10.3945/ajcn.2010.29649.
15. Kiskini, A.; Hamer, H.M.; Wall, B.T.; Groen, B.B.; de Lange, A.; Bakker, J.A.; Senden, J.M.; Verdijk, L.B.; van Loon, L.J. The muscle protein synthetic response to the combined ingestion of protein and carbohydrate is not impaired in healthy older men. *Age (Dordrecht, Netherlands)* **2013**, *35*, 2389–2398, doi:10.1007/s11357-013-9522-2.
16. Gorissen, S.H.; Burd, N.A.; Hamer, H.M.; Gijsen, A.P.; Groen, B.B.; van Loon, L.J. Carbohydrate coingestion delays dietary protein digestion and absorption but does not modulate postprandial muscle protein accretion. *The Journal of clinical endocrinology and metabolism* **2014**, *99*, 2250–2258, doi:10.1210/jc.2013-3970.
17. Sheffield-Moore, M.; Paddon-Jones, D.; Sanford, A.P.; Rosenblatt, J.I.; Matlock, A.G.; Cree, M.G.; Wolfe, R.R. Mixed muscle and hepatic derived plasma protein metabolism is differentially regulated in older and younger men following resistance exercise. *American journal of physiology. Endocrinology and metabolism* **2005**, *288*, E922–929, doi:10.1152/ajpendo.00358.2004.
18. Kumar, V.; Selby, A.; Rankin, D.; Patel, R.; Atherton, P.; Hildebrandt, W.; Williams, J.; Smith, K.; Seynnes, O.; Hiscock, N.; et al. Age-related differences in the dose-response relationship of muscle protein synthesis to resistance exercise in young and old men. *The Journal of physiology* **2009**, *587*, 211–217, doi:10.1113/jphysiol.2008.164483.
19. Mayhew, D.L.; Kim, J.S.; Cross, J.M.; Ferrando, A.A.; Bamman, M.M. Translational signaling responses preceding resistance training-mediated myofiber hypertrophy in young and old humans. *Journal of applied physiology (Bethesda, Md. : 1985)* **2009**, *107*, 1655–1662, doi:10.1152/japplphysiol.91234.2008.
20. Fry, C.S.; Drummond, M.J.; Glynn, E.L.; Dickinson, J.M.; Gundermann, D.M.; Timmerman, K.L.; Walker, D.K.; Dhanani, S.; Volpi, E.; Rasmussen, B.B. Aging impairs contraction-induced human skeletal muscle mTORC1 signaling and protein synthesis. *Skeletal Muscle* **2011**, *1*, 11, doi:10.1186/2044-5040-1-11.
21. Kumar, V.; Atherton, P.J.; Selby, A.; Rankin, D.; Williams, J.; Smith, K.; Hiscock, N.; Rennie, M.J. Muscle protein synthetic responses to exercise: effects of age, volume, and intensity. *The journals of gerontology. Series A, Biological sciences and medical sciences* **2012**, *67*, 1170–1177, doi:10.1093/gerona/gls141.
22. Brook, M.S.; Wilkinson, D.J.; Mitchell, W.K.; Lund, J.N.; Phillips, B.E.; Szewczyk, N.J.; Greenhaff, P.L.; Smith, K.; Atherton, P.J. Synchronous deficits in cumulative muscle protein synthesis and ribosomal biogenesis underlie age-related anabolic resistance to exercise in humans. *The Journal of physiology* **2016**, *594*, 7399–7417, doi:10.1113/jp272857.
23. Reitelseder, S.; Bülow, J.; Holm, L. Divergent Anabolic Response to Exercise in Young and Older Adult Men-Dependency on Time Frame of Measurement. *The journals of gerontology. Series A, Biological sciences and medical sciences* **2021**, *76*, 996–999, doi:10.1093/gerona/glab040.
24. Sheffield-Moore, M.; Yeckel, C.W.; Volpi, E.; Wolf, S.E.; Morio, B.; Chinkes, D.L.; Paddon-Jones, D.; Wolfe, R.R. Postexercise protein metabolism in older and younger men following moderate-intensity aerobic exercise. *American journal of physiology. Endocrinology and metabolism* **2004**, *287*, E513–522, doi:10.1152/ajpendo.00334.2003.
25. Koopman, R.; Verdijk, L.; Manders, R.J.; Gijsen, A.P.; Gorselink, M.; Pijpers, E.; Wagenmakers, A.J.; van Loon, L.J. Co-ingestion of protein and leucine stimulates muscle protein synthesis rates to the same extent in young and elderly lean men. *The American journal of clinical nutrition* **2006**, *84*, 623–632, doi:10.1093/ajcn/84.3.623.
26. Drummond, M.J.; Dreyer, H.C.; Pennings, B.; Fry, C.S.; Dhanani, S.; Dillon, E.L.; Sheffield-Moore, M.; Volpi, E.; Rasmussen, B.B. Skeletal muscle protein anabolic response to resistance

exercise and essential amino acids is delayed with aging. *Journal of applied physiology (Bethesda, Md. : 1985)* **2008**, *104*, 1452–1461, doi:10.1152/japplphysiol.00021.2008.
27. Durham, W.J.; Casperson, S.L.; Dillon, E.L.; Keske, M.A.; Paddon-Jones, D.; Sanford, A.P.; Hickner, R.C.; Grady, J.J.; Sheffield-Moore, M. Age-related anabolic resistance after endurance-type exercise in healthy humans. *FASEB journal : official publication of the Federation of American Societies for Experimental Biology* **2010**, *24*, 4117–4127, doi:10.1096/fj.09-150177.
28. Marshall, R.N.; Morgan, P.T.; Smeuninx, B.; Quinlan, J.I.; Brook, M.S.; Atherton, P.J.; Smith, K.; Wilkinson, D.J.; Breen, L. Myofibrillar Protein Synthesis and Acute Intracellular Signaling with Elastic Band Resistance Exercise in Young and Older Men. *Medicine and science in sports and exercise* **2023**, *55*, 398–408, doi:10.1249/mss.0000000000003061.
29. Symons, T.B.; Sheffield-Moore, M.; Mamerow, M.M.; Wolfe, R.R.; Paddon-Jones, D. The anabolic response to resistance exercise and a protein-rich meal is not diminished by age. *The journal of nutrition, health & aging* **2011**, *15*, 376–381, doi:10.1007/s12603-010-0319-z.
30. Atherton, P.J.; Kumar, V.; Selby, A.L.; Rankin, D.; Hildebrandt, W.; Phillips, B.E.; Williams, J.P.; Hiscock, N.; Smith, K. Enriching a protein drink with leucine augments muscle protein synthesis after resistance exercise in young and older men. *Clinical nutrition (Edinburgh, Scotland)* **2017**, *36*, 888–895, doi:10.1016/j.clnu.2016.04.025.
31. Moro, T.; Brightwell, C.R.; Deer, R.R.; Graber, T.G.; Galvan, E.; Fry, C.S.; Volpi, E.; Rasmussen, B.B. Muscle Protein Anabolic Resistance to Essential Amino Acids Does Not Occur in Healthy Older Adults Before or After Resistance Exercise Training. *The Journal of nutrition* **2018**, *148*, 900–909, doi:10.1093/jn/nxy064.
32. Horwath, O.; Moberg, M.; Hodson, N.; Edman, S.; Johansson, M.; Andersson, E.; van Hall, G.; Rooyackers, O.; Philp, A.; Apró, W. Anabolic Sensitivity in Healthy, Lean, Older Men Is Associated With Higher Expression of Amino Acid Sensors and mTORC1 Activators Compared to Young. *J Cachexia Sarcopenia Muscle* **2025**, *16*, e13613, doi:10.1002/jcsm.13613.
